# *Drosophila* mitochondrial topoisomerase III alpha affects the aging process via maintenance of mitochondrial function and genome integrity

**DOI:** 10.1186/s12929-016-0255-2

**Published:** 2016-04-12

**Authors:** Han-Zen Tsai, Ren-Kuo Lin, Tao-Shih Hsieh

**Affiliations:** Institute of Cellular and Organismic Biology, Academia Sinica, No. 128, Academia Road, Sec. 2, Nangang, Taipei 11529 Taiwan; Graduate Institute of Life Sciences, National Defense Medical Center, No. 161, Minquan East Road, Sec. 6, Neihu, Taipei 11490 Taiwan

**Keywords:** Mitochondrial topoisomerase III alpha (mtTop3α), Mitochondria dysfunction, Mitochondrial DNA deletion, Aging

## Abstract

**Background:**

Mitochondria play important roles in providing metabolic energy and key metabolites for synthesis of cellular building blocks. Mitochondria have additional functions in other cellular processes, including programmed cell death and aging. A previous study revealed *Drosophila* mitochondrial topoisomerase III alpha (Top3α) contributes to the maintenance of the mitochondrial genome and male germ-line stem cells. However, the involvement of mitochondrial Top3α in the mitochondrion-mediated aging process remains unclear. In this study, the M1L flies, in which Top3α protein lacks the mitochondrial import sequence and is thus present in cell nuclei but not in mitochondria, is used as a model system to examine the role of mitochondrial Top3α in the aging of fruit flies.

**Results:**

Here, we reported that M1L flies exhibit mitochondrial defects which affect the aging process. First, we observed that M1L flies have a shorter life span, which was correlated with a significant reduction in the mitochondrial DNA copy number, the mitochondrial membrane potential, and ATP content compared with those of both wildtype and transgene-rescued flies of the same age. Second, we performed a mobility assay and electron microscopic analysis to demonstrate that the locomotion defect and mitophagy of M1L flies were enhanced with age, as compared with the controls. Finally, we showed that the correlation between the mtDNA deletion level and aging in M1L flies resembles what was reported in mammalian systems.

**Conclusions:**

The results reported here demonstrate that mitochondrial Top3α ablation results in mitochondrial genome instability and its dysfunction, thereby accelerating the aging process.

## Background

In addition to their well-known functions in metabolism, mitochondria also play an integral role in programmed cell death and in the aging process [1, 2]. There are complicated genetic interactions, which are important for maintaining cellular functions, between nuclei and mitochondria [3–6]. Among the approximately 1500 proteins necessary for assembling a mitochondrion, all but 13 of them are encoded by the nuclear genome [7, 8]. Most of the animal mitochondrial genomes possess a compact and circular structure. For example, the *Drosophila* mitochondrial genome, being a larger member in this family, is a circular molecule of 19.5 kb [9]. The mitochondrial genome encodes several RNA components, 2 rRNAs and 22 tRNAs, necessary for the intra-mitochondrial translational machinery, which are responsible for making the peptides encoded by the mitochondrial genome. These thirteen proteins of mitochondrial origin are components in the oxidative phosphorylation chain [5, 7, 10], including complexes I, III and IV, transferring the electrons to molecular oxygen and pumping protons to the inter-membrane space, and complex V (ATP synthase) for utilizing the potential energy of the proton gradient to generate ATP [5]. It has been speculated that the continuing synthesis of these thirteen proteins inside mitochondria is critical not only for ATP synthesis but also for the integrity of mitochondria (including maintaining a positive electrochemical potential in the inter-membrane space vs matrix) [11]. Therefore, normal mitochondrial function requires maintaining the integrity of the mitochondrial genome throughout life.

Replication and repair of the mitochondrial genome are performed by proteins encoded by the nuclear genome [10]. The basic DNA synthesis and RNA transcriptional machines are distinct from those used for chromosomal counterparts in the nucleus, with one of the notable exceptions being topoisomerase IIIα (Top3α), which is present in both the nuclear and mitochondrial compartments. Top3α has two in-frame initiating methionines at its amino-terminus and a mitochondrial import sequence between the first and second methionine. Therefore, the methionine used for translational initiation determines whether the products are destined to enter the mitochondria or nuclei.

We previously exploited the genetic amenability of *Drosophila* to demonstrate the presence, as well as the requirement, of Top3α for maintaining the mitochondrial genome [12]. We generated a fly strain, M1L, in which the first methionine of Top3α was mutated to leucine. Both male and female M1L flies, which lack Top3α in mitochondria, show fertility defects. The fertility defects were found to be associated with both reduced mitochondrial DNA (mtDNA) copy number and decreased ATP content. In this study, we examined the biological dysfunction and abnormal phenotype of adult flies with mitochondrial Top3α deficiency.

## Methods

### *Drosophila* strains

The null *top3α Drosophila* strains and *Top3α* transgenic lines were generated previously [12]. The *Top3α*-rescued (*top3α*^*54*^*;Top3α-YFP*) and M1L-YFP (*top3α*^*54*^*;M1L-YFP*) flies were made by crossing *Top3α* deletion heterozygous flies (*top3α*^*54*^*/CyO*) with Top3α transgenic flies (*+/CyO;Top3α-YFP*) and M1L transgenic flies (*+/CyO;M1L-YFP*), respectively. All flies were raised at 25 °C under a 12 h light :12 h dark cycle on standard fly food.

### Life span analysis

Flies were kept at a density of 20 flies per vial after eclosion. All flies were kept in an incubator under a 12 h light:12 h dark cycle at 25 °C. Flies were transferred to fresh vials every 2 days, and viability was recorded at the time of vial transfer.

### Quantitative PCR

Fifty flies of the indicated genotype and age were homogenized in a buffer containing 10 mM Tris (pH 8.0), 1 mM EDTA, and 25 mM NaCl. Homogenates were treated with proteinase K (0.15 mg/ml) at 65 °C for 15 min, followed by incubation at 95 °C for 15 min. Samples were cleared by centrifugation at 12,000Xg for 5 min at 4 °C, and supernatant was analyzed by quantitative-PCR with gene-specific primer sets for cytochrome c oxidase subunit II (GATGTTGATAACCGAGTAGTTTTACCT and AAGCAGTACTGTTCAAGA-ATGAAT) and *rosy* (GGTGGTGAGCCTGTTCTTCAAG and ACTGGTGTGT-GGAATGTCTCGG) as a readout for mtDNA and as an internal control, respectively.

### Mitochondrial membrane potential assay

Mitochondria were prepared by homogenizing 50 adult flies in buffer D (5 mM Tris, pH 8.0, 0.5 mM EDTA) before centrifugation at 500Xg for 30 s at 4 °C. Mitochondrial membrane potential was determined using the JC-1 Kit (T3168, Invitrogen). Aggregations of JC-1 were examined based on their emission at 590 nm, as measured using a PerkinElmer counter. Mitochondrial membrane potential was normalized to total protein amount.

### ATP assay

Whole-body homogenized lysate (from 50 flies) was prepared in buffer D and cleared by centrifugation (500Xg, 30 s, 4 °C). ATP levels were determined using the ATP Bioluminescent Assay Kit (V6072, Promega) according to the manufacturer’s protocol. Photon emission was determined using a PerkinElmer counter. ATP contents were normalized to total protein amount.

### Climbing assay

Mobility was analyzed by placing 30 adults in a fresh vial (23 mm X 95 mm) and allowing them to accommodate to the new environment for 30 min. Flies were then gently tapped to the bottom of the vial and the number of flies reaching the top one-third of the vial after 20 s was measured [13, 14]. Motor performance was calculated in this manner once a week. Each experiment included 150 males and females of each genotype and the data were analyzed to determine statistical significance.

### Immunostaining

Tissue dissection and immunostaining were performed as described previously with modifications [15]. In brief, adult ventral abdominal segments were dissected along the dorsal midline in PBS (phosphate-buffered saline), and were then fixed in PBS containing 4 % paraformaldehyde at room temperature for 10 min. After blocking (PBS plus 3 % BSA and 3 % normal goat serum), samples were incubated with the antibodies overnight at 4 °C followed by washing in PBST (PBS with 0.3 % Triton-X 100). All samples were mounted with Prolong® Gold antifade reagent (P36935, Molecular Probes). The following antibodies were used: Goat anti-HRP (123-605-021, Jackson Immuno); Mouse anti-Brp (5B6, Hybridoma Bank, University of Iowa); Goat anti-mouse Ig-Alexa 488 (A-11001, Molecular Probes).

### Electron microscopy

*Drosophila* indirect flight muscles were dissected in ice-cold PBS and fixed in 4 °C in 0.1 M sodium cacodylate buffer (pH 7.4) with 2.5 % glutaraldehyde and 4 % paraformaldehyde for 4 h. Samples were post-fixed in 0.1 M sodium cacodylate buffer containing 1 % OsO_4_ for 2 h, and were then embedded in Spurr’s resin (EMS 550) for 3 days at 37 °C after dehydration. Ultrathin sections (50 nm) were cut and stained with 4 % uranyl acetate and 3 % lead citrate before being examined using a Hitachi H7000 transmission electron microscope.

### Next-generation sequencing of mtDNA

Purification of mtDNA from flies was performed as previously described [16]. Purified mtDNA was fragmented to an average size of 300 bps by sonication. The sequences of mtDNA small fragments were determined with next-generation sequencing (NGS). Deletions in mtDNA were defined by alignment of the unmapped reads, with degenerative parameters to reduce stringency against a *Drosophila* mitochondrial DNA reference sequence (GeneBank: U37541.1), using integrative genomics viewer (IGV) software. Deletion junctions within a coverage ranging from 1.1 to 13.3 kbps were selected. The mtDNA deletion frequency was calculated by dividing the number of the characterized deletion reads by that of total reads.

## Results

### Depletion of mitochondrial Top3α shortens lifespan in *Drosophila*

**Fig. 1:**
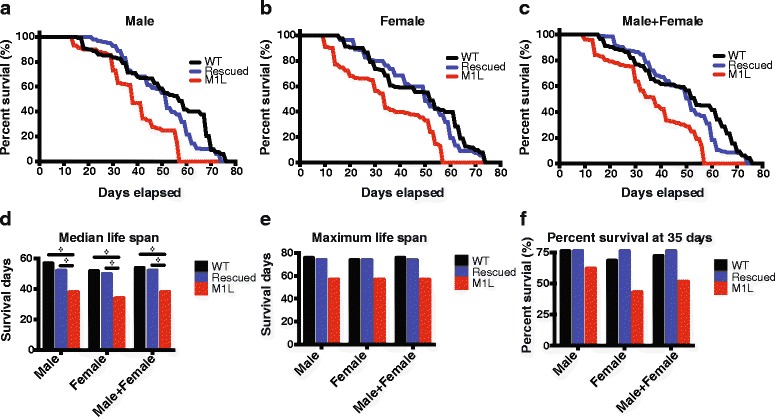
Depletion of Top3α in mitochondria shortens the lifespan of *Drosophila*. The lifespans of WT (wildtype), rescued, and M1L (**a**) male, (**b**) female, and (**c**) mixed populations were measured. Median lifespan (**d**), maximum lifespan (**e**), and 35-day survival rate (**f**) are shown for each group. **d**) also shows the log-rank test demonstrating the statistically significant difference in life span between mutant and control flies (❖for *P* < 10^-12^ with log-rank test). The total number of flies was 200 for both male and female groups, and 400 for the mixed group

To determine the effect of ablation of mitochondrial Top3α in *Drosophila* adults, we measured the life span of M1L mutant (*top3α*^*54*^*;M1L-YFP*), transgene-rescued (*top3α*^*54*^*;Top3α-YFP*) and wildtype (Oregon R) flies. We observed that M1L flies showed an early decline in survival rate and a shortened maximal survival time, as compared to wildtype and rescued flies (Fig. 1a-c). From these survival curves, we were also able to demonstrate differences in median life span, maximum life span, and 35-day survival (Fig. 1d-f). The statistical significance of the differences in their median life span was validated by log-rank test (Fig. 1d). We then cultured the male and female flies together at a ratio of 1 to 1. In this mixed group, the M1L flies exhibited a reduction in median life span of more than 20 %, as compared with the other two strains.

The maximal life span results were similar to those of median life span. The male and mixed M1L flies exhibited a 25 and 23 % reduction when compared with wildtype and rescued flies, respectively, while female M1L flies showed a 23 % decrease as compared with both wildtype and rescued flies (Fig. 1e). Additionally, no significant difference in survival was observed before the first week (Fig. 1a-c). However, by 5 weeks old, M1L flies of all three groups (males, females and mixed) showed a significant reduction in survival rate (Fig. 1f). Specifically, we observed reductions of 20 and 21 % for the male group; 33 and 43 % for the female group; and 26 and 31 % for the mixed group, as compared with the wildtype and rescued strains, respectively. Thus, these data indicate that loss of mitochondrial Top3α caused premature aging in *Drosophila*.

### Mitochondrial Top3α Is required for mitochondrial genome maintenance and ATP generation

**Fig. 2:**
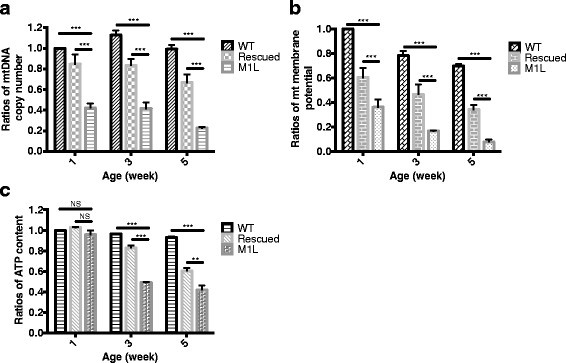
Mitochondrial Top3α is required for mitochondrial genome maintenance and energy generation. **a** M1L flies exhibit lower mtDNA copy numbers than controls at the ages examined. **b** The decrease of mitochondrial membrane potential is accelerated in M1L flies compared to wildtype and rescued flies. **c** ATP content in M1L flies declines more rapidly with age when compared to wildtype and rescued counterparts. Two-way ANOVA with Turkey’s multiple tests was used for statistical analysis. (NS = not significant, ***P < 0.001, **P < 0.01, *P < 0.05)

Mutant flies with a deletion of mitochondrial Top3α exhibited shortened life span and earlier decline of life span (Fig. 1a-c), and it is possible that mitochondrial Top3α deletion mediates its effect on lifespan through disruption of mitochondrial function. To examine the requirement of Top3α in the maintenance of the mitochondrial genome, we measured the mitochondrial DNA copy number in adult flies of different genotypes and ages by quantitative PCR. Since the survival rate of M1L flies is not different to that of WT flies at younger ages (Fig. 1a-c and f), we decided to monitor mitochondrial DNA content and mitochondria functions of 1, 3 and 5-week-old flies. We found that mitochondrial DNA copy number in the M1L flies was decreased 2.38 and 2-fold at 1 week of age compared with the wildtype and transgene-rescued flies, respectively. By 5 weeks of age, the decrease of mtDNA in M1L flies was enhanced by 4.5 and 3-fold, as compared to the respective controls (wildtype and rescued flies) (Fig. 2a).

We also determined the mitochondrial membrane potential of the mutant flies by monitoring JC-1 aggregation [17]. As shown in Fig. 2b, the mitochondrial membrane potential of M1L mutant flies was 3.4, 4.6, and 5.3-fold lower than that of wildtype flies at 1-, 3-, and 5-week-old, respectively. As compared with the rescued flies, the membrane potential in M1L mutants was 2, 2.4, and 5.3-fold lower at 1, 3 and 5 weeks old, respectively. Similar to the premature decline in survival rate (Fig. 1a-c), M1L flies also exhibited a faster decrease in mitochondrial membrane potential. Specifically, while membrane potential was reduced with age (comparing 5- to 1-week-old flies) by 30 % in wildtype and 44 % in rescued flies, it was reduced by 77 % in M1L flies.

Since the mitochondrial membrane potential is required for ATP production by ATP synthase, we also measured ATP content as an endpoint for ATP generation. Compared with the wildtype and rescued flies, the ATP content of the 1-week-old M1L flies was decreased by 1.05 and 1.07-fold, respectively, while in the 5-week-old M1L flies, the ATP content was decreased by 2.19 and 1.44-fold (Fig. 2c). Consistent with these findings, the age-dependent decrease of ATP content was more apparent in M1L flies (56 %) than that in either wildtype (7 %) or rescued flies (40 %). Our results indicate that mitochondrial Top3α deficiency causes marked declines in mitochondrial DNA copy number, mitochondrial membrane potential, and ATP content with age.

### Mitochondrial Top3α depletion causes mobility defects

In order to further explore the premature aging phenotype in M1L flies, we examined the performance of flies of different ages (1 to 5 weeks old) in a locomotion assay [14].

**Fig. 3:**
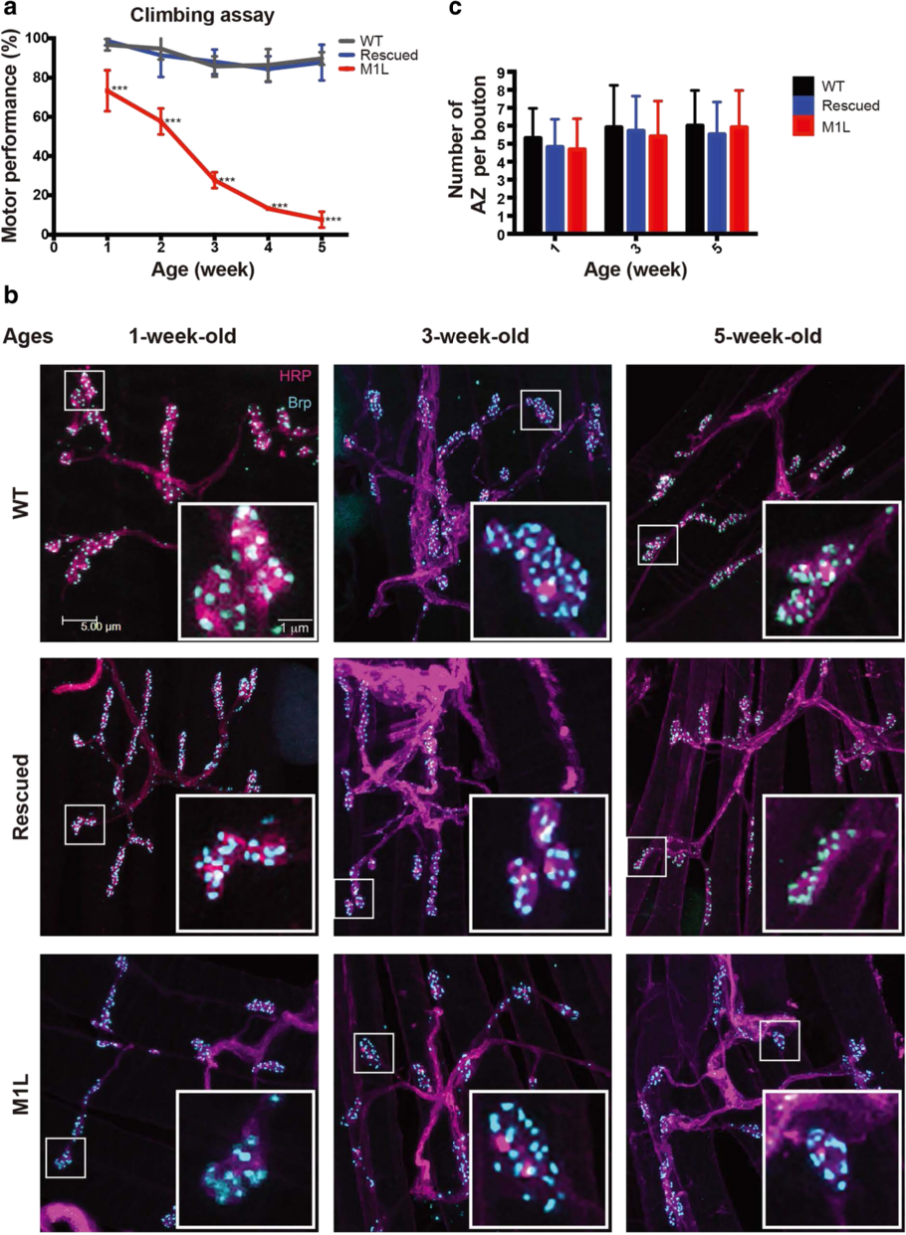
Mitochondrial Top3α depletion causes mobility defects in *Drosophila*. **a** Mobility of M1L adults decreases rapidly with age as shown by climbing assay. Each experiment included 300 adults of each genotype at the indicated age. One-way ANOVA with Tukey’s multiple test was performed to compare M1L with WT (wildtype) and rescued flies (***P < 0.001). **b** The muscle of Lawrence (MOL) from wildtype, rescued, and M1L adult males was dissected and stained with anti-HRP (neuron, magenta) and anti-Brp (active zone, cyan) antibodies. **c** The numbers of active zones (AZ) per bouton (*n* = 42) were similar for all samples examined in this study

Although similar locomotion behavior was retained in the wildtype and the rescued flies between 1 and 5 weeks of age, the climbing activity of the M1L flies steadily declined during the same period (Fig. 3a), indicating that mitochondrial Top3α deficiency affects mobility in *Drosophila*.

The results of the climbing assay may reflect cellular energy levels, but it could also indicate defects in neuronal development. To examine whether neuronal defects exist in the mutants, we examined the morphology of the neuromuscular junction (NMJ) by immunostaining. We observed that zygotic elimination of mitochondrial Top3α did not significantly affect NMJ morphology (Fig. 3b). NMJ formation on the muscle fiber and active zone assembly at the bouton are not significantly affected in M1L flies. This observation suggests that deletion of mitochondrial Top3α does not alter the development of motor neurons. We did not observe any morphological changes, such as the appearance of satellite boutons or a reduction in the size of a bouton, in the NMJ of the threes genotypes examined (Fig. 3b and inserts). Furthermore, the overall structure of the NMJ did not noticeably change between 1 and 5 weeks of age in any of the three genotypes examined. Similarly, no changes in the numbers of active zones per bouton were observed between different ages or genotypes (Fig. 3c). These results suggest that the development of motor neurons is not compromised by depleting mitochondrial Top3α, and thus the climbing defects are likely caused by decreased ATP production.

### M1L flies exhibit elevated levels of mitochondrial degeneration

**Fig. 4:**
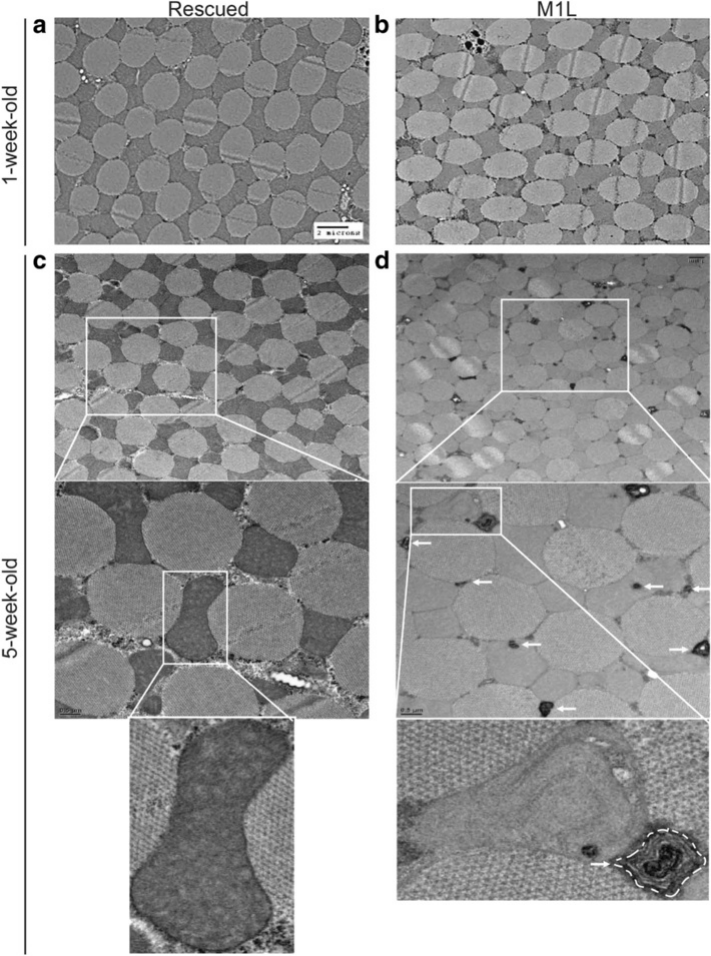
Membrane aggregates from degenerated mitochondria are observed in the indirect flight muscle of M1L flies. EM images of the indirect flight muscles from rescued (**a**, **c**) and M1L (**b**, **d**) flies at 1 (**a**, **b**) and 5 weeks old (**c**, **d**). Magnified views of the boxed regions are shown below each panel. Arrows indicate the degenerated mitochondria

We proceeded to use electron microscopy to examine whether the absence of mitochondrial Top3α affected the morphology of mitochondria. Removal of Top3α from mitochondria did not alter mitochondrial morphology in 1-week-old flies (Fig. 4a-b). Additionally, no significant difference in the overall structure of muscle fibers was observed in the mutant, suggesting that the development of muscle is normal. However, by 5 weeks of age, the samples from M1L flies contained more electron-dense particles, possibly membrane aggregates from degenerated mitochondria, than those from rescued flies (compare Fig. 4c vs d, white arrows mark the membrane aggregates). According to previous studies [18–20], these aggregates are the debris derived from the products of mitophagy of dysfunctional mitochondria. Upon loss of mitochondrial membrane potential, the E3 ubiquitin ligase Parkin translocates to the mitochondrial outer-membrane [21, 22], thereby targeting the dysfunctional and damaged mitochondrion for degradation by autophagosomes. Thus, our observation of mitochondrial membrane debris in M1L flies is consistent with the observed loss of mitochondrial membrane potential during aging (Fig. 2b).

### M1L flies exhibit age-dependent increases in the deletion of mtDNA

**Fig. 5:**
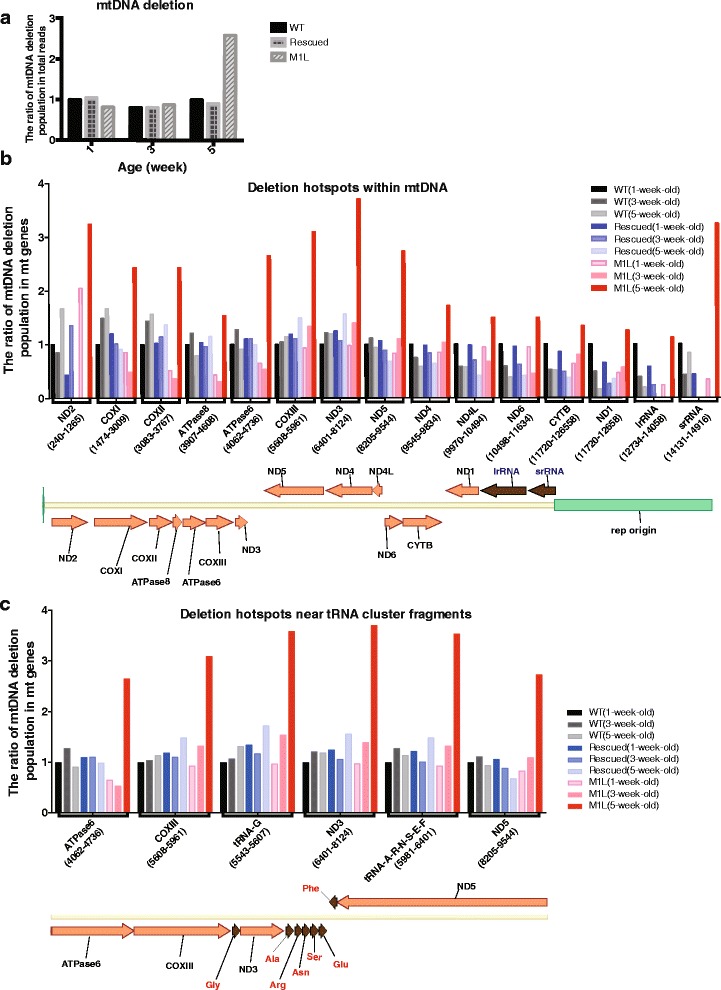
Mitochondrial Top3α deficiency leads to mitochondrial genome instability. **a** NGS analysis revealed elevated mtDNA deletions in 5-week-old M1L flies. **b** The frequency of DNA deletions in mtDNA genes. All values were normalized to those of 1-week-old wildtype. The lower panel is a schematic diagram showing the relative location of genes on mtDNA. Arrows above the line indicate genes that are encoded on the light strand, while arrows below the line indicate genes on the heavy strand. Protein- and RNA-coding genes are marked by orange and brown arrows, respectively. **c** Detailed features of the deletion hotspot surrounding the tRNA cluster (brown arrows with amino acids marking each tRNA species)

The results described above indicate that mitochondrial Top3α depletion induces premature aging in flies. Previous studies have reported that mtDNA deletions accumulate in various mammalian tissues during aging [23–27], and we therefore proceeded to use next-generation sequencing to determine if premature aging in M1L flies is associated with mtDNA deletion. We chose to examine mtDNA deletions in the range of (1100–13,300 bp) among these three genotypes at various ages. For wildtype and rescued flies, no significant differences in the levels of mtDNA deletion were observed between 1 and 5 weeks of age. However, while deletion levels were similar between 1 and 3 weeks of age in M1L flies, a 2.59-fold increase was observed when these mutant flies were 5-week-old (Fig. 5a).

We then calculated the deletion levels of each mitochondrial gene, by normalizing mtDNA deletion frequency to that of 1-week-old wildtype flies. Apparent increases (over 2-fold) in the levels of mtDNA deletion were observed for some genes in 5-week-old M1L flies. Deletion hotspots were identified in the region from 4062 nt to 9544 nt, which includes the ATPase6, COIII, ND3, and ND5 genes. Another hotspot was identified in the sequences neighboring the AT-rich region at the replication origin for heavy strand synthesis, which encodes ND2-COXII and srRNA (Fig. 5b). We further analyzed the region from 4062 to 9544 nt in greater detail (Fig. 5c), revealing that this hotspot is located in the vicinity of the NADH3 gene and tRNA clusters. These tRNA clusters were shown to be the replication origin for the light strand DNA synthesis in mammalian mtDNA [8, 28]. Our results indicate that mtDNA deletions increase more rapidly with age in M1L flies, which could cause the observed mitochondrial dysfunction and early decline in life span in the absence of mitochondrial Top3α.

## Discussion

Aging is a process through which physiological functions gradually decline and the risk of morbidity and mortality steadily increases. It has been previously shown that aging is associated with major changes in mitochondrial functions and genome, including a reduction of oxidative phosphorylation (OXPHOS), increased deterioration of mitochondrial structure, and increased levels of mtDNA deletions [29]. Our findings here reveal key roles for Top3α in mitochondrial functions, maintenance of mitochondrial genome integrity, and the progression of aging.

It has been shown that mtDNA copy number is negatively correlated with age [30]. Decreased mtDNA content is also linked to the decline of mitochondrial function [31]. In the present study, we demonstrated a sharp decrease of mtDNA levels with age in flies lacking mitochondrial Top3α (Fig. 2a). Additionally, mitochondrial functions in M1L flies are diminished as compared to those of wildtype and rescued flies (Fig. 2b and c). It has been shown that a reduction of mitochondrial activity will not occur until a considerable level (~60 %) of mtDNA deletion has been reached [32]. This so-called “threshold effect” varies between different types of cells. Loss of mitochondrial Top3α results in both accumulation of mtDNA deletions and a concomitant decrease of mtDNA copy number (Fig. 2a and 5a). Therefore, multiple changes in the mitochondrial genome may lead to the observed mitochondrial dysfunction in M1L flies.

Our results indicate that a deficiency in mitochondrial Top3α plays a role in accelerating mtDNA genome instability and progression of aging in mutant flies (Fig. 5a). In humans, the most prevailing deletions are mapped in the proximity of replication origins for both the heavy and light strand [33]. Interestingly, our results from NGS analysis demonstrated that the hotspots for mtDNA deletion in *Drosophila* are located adjacent to the heavy strand origin, and also to the tRNA clusters, which are possibly the replication origin for the light strand (Fig. 5b and c). The deletion frequency also increases as the mtDNA copy number decreases in the M1L mutant flies (Fig. 5a and 2a). PCR-based assays and Southern blot analysis have demonstrated that mtDNA deletions accumulate with age in *Drosophila* [34]. However, such analysis did not provide any detailed information on the deletion frequency or the sequence boundaries for deletions. Here, we examined mtDNA deletions with NGS analysis, which not only allowed us to assess mtDNA deletion frequency, but to also examine deletion hotspots at the sequence level. According to our NGS data, there are mtDNA deletions in genes encoding COXI, II, and III, ND2, 3, and 5, and ATPase6 in 5-week-old M1L flies. These genes code for subunits of cytochrome c oxidase (Complex IV), NADH dehydrogenase (Complex I), and ATP synthase (Complex V), respectively. Therefore, by 5 weeks of age, the mutant flies will not be able to generate the requisite proton gradient (due to defects in complex I and IV) or produce ATP (due to defects in complex V), leading to a decrease in the cellular levels of ATP, as observed here.

Most mitochondrial genomes are circular and consist of covalently closed DNA [35]. Essentially all fundamental processes, including replication and transcription, that take place on circular dsDNA require the strand passage activities of DNA topoisomerases [36, 37]. These enzymes can provide a swivel for relieving torsional stress generated by the advancement of replication or transcription forks, and to segregate the interlocked daughter chromosomes after finishing replication.

There are two well-documented mitochondrial topoisomerases in mammalian cells, Top1mt (mitochondrial topoisomerase I) [38] and Top3α [39]. Top1mt is encoded in the nuclear genome and imported into mitochondria due to the mitochondrial import sequence at its N-terminus [38]. Although Top1mt is not essential for mouse viability and development, Top1mt-knockout mice possess mtDNA with an increased level of negative supercoiling [40]. While Top3α has been demonstrated to be essential for mouse embryonic development [41], its function in mitochondria biogenesis is unclear. There is no Top1mt in *Drosophila*, and Top3α is the only observed topoisomerase present in mitochondria [12]. For a type IA topoisomerase like Top3α, one may expect it functions as a swivel to resolve entanglement generated during transcription and replication. However, the segregation of replicated and interlocked daughter chromosomes is usually reserved for a type II enzyme. In the absence of a clearly-documented type II enzyme in *Drosophila* mitochondria, how is mtDNA replicated and segregated in this organism? We believe that the special mode of replication for mtDNA may mean that a type IA enzyme can resolve the catenated circular molecules, as they contain single-stranded gaps [42, 43].

The prevailing model of mtDNA replication is termed asymmetric synthesis by strand displacement [44, 45]. It is expected that replication through the strand displacement model would generate extensive single stranded regions, since the synthesis of the light strand lags behind that of the heavy strand, thus leaving a long single-stranded gap in the so-called D-loop replication intermediate [46]. Furthermore, there appear to be multiple events of RNA priming and replication initiation, thus leaving behind small single-strand gaps as well [47, 48]. These single-stranded gaps in the interlocked circular molecules can serve as a strand passage site for type IA enzymes, as was demonstrated for *E. coli* replication intermediates [49].

Our hypothesis that Top3α can multitask to serve as a swivel to remove torsional stress and to segregate interlocked circular DNAs also implies that its removal would have deleterious effects on mtDNA maintenance. Stalled replication/transcription forks are vulnerable to DNA damage, and the tearing of interlocked DNA rings when an organelle attempts to undergo a fission process can generate DNA breaks. The repair of such DNA damage and breaks may lead to decreases in DNA copy number and deletions at multiple loci around the genome, as we have observed here. Our results here therefore provide an important insight into the role of Top3α in mtDNA maintenance and mitochondrial function.

## Conclusions

Our results indicate that premature aging and mobility defects in M1L flies arise from defects in mitochondria in the absence of mitochondrial Top3α, and are associated with the accumulation of mtDNA deletions.

### Ethics approval and consent to participate

Not applicable.

### Consent for publication

Not applicable.

## Abbreviations

Top3α: topoisomerase III alpha
Top1mt: mitochondrial topoisomerase I
mtDNA: mitochondrial DNA
NGS: next generation sequencing
NMJ: neuromuscular junction
ND: NADH dehydrogenase
COX: cytochrome c oxidase
CYTB: cytochrome b oxidase
ATPase: ATP synthase
srRNA: small ribosomal RNA
lrRNA: large ribosomal RNA
